# Genetic Polymorphisms of Glutathione S-Transferases T1 (*GSTT1*) and M1 (*GSTM1*) in Iranian Mandaeans Population

**Published:** 2019-09

**Authors:** Fariba BOROUMAND, Mahdis ZARGHAMI, Mostafa SAADAT

**Affiliations:** Department of Biology, College of Sciences, Shiraz University, Shiraz, Iran

## Dear Editor-in-Chief

Glutathione S-transferases (GSTs; EC: 2.5.1.18) are ubiquitous enzymes which contribute to the protection against a varied range of harmful chemicals including carcinogens, drugs, pesticides and environmental pollutants (1). The *GSTM1* (OMIM: 138350) and *GSTT1* (OMIM: 600436) belong to mu and theta classes of the GST superfamily. Null alleles in these two genes have been well defined. The null alleles represent deletions of the corresponding genes and result in a loss of enzymatic activity (1). Studies indicated that these polymorphisms were associated with complex multifactorial trials (1–3). The *GSTT1* and *GSTM1* null genotypes showed variations between different ethnic groups (4–6).

Numerous studies on genetic structure of Iranian populations indicate that Iran has one of the most heterogeneous populations of the world. Mandaean (a small and closed ethno-religious) community is one of the Iranian sub-populations. They strongly have a dualistic worldview. They migrated from Jordan/Palestine areas to southern Iraq and south-west Iran areas about 20 centuries ago. Mandaeans language belongs to Eastern Aramaic language (7).

Very recently the prevalence of consanguinity among Iranian Mandaeans living in Khuzestan Province was reported (8). However, there is no published data about genetic structure of Mandaean population. Therefore, the present study was carried out to determine the frequency of the *GSTT1* and *GSTM1* null genotypes in Iranian Mandaeans.

The total study subjects consisted of 119 (50 males, 69 females) healthy Mandaeans living in Khuzestan Province (south-west Iran) in 2016.

The present study was approved by the Ethics Committee of Shiraz University. Written informed consent was obtained from each participant. The study was carried out in accordance with the Code of Ethics of the World Medical Association (Declaration of Helsinki) for Ethical Principles of Medical Research Involving Human Subjects.

Genomic DNA was extracted from whole blood using a salting-out method. Genetic polymorphisms for *GSTT1* and *GSTM1* were determined by multiplex PCR in which β-globin gene was co-amplified as an internal control (5, 6).

Since there was no statistically significant difference between gender groups for the study polymorphisms, the sex groups were pooled. The null genotypes of *GSTT1* and *GSTM1* was 16.0% (95% CI: 9.3%–22.7%) and 76.5% (95% CI: 68.7%–84.3%), respectively. The *GSTM1* null genotype in other Iranian ethnic groups has been reported about 41%–58% (5, 6), which revealed highly significant differences with Iranian Mandaeans.

About 20 century ago, Mandaeans migrated from Jordan/Palestine areas to Iraq and Iran (7). Therefore, their gene pool was separated from their origins for about 20 centuries. During this period, evolutionary forces might have some effects on Mandaeans’ gene pool. The frequency of the *GSTM1* null genotype among Jordanian, Palestinian, Ashkenazi Jews and non-Ashkenazi Jews was 27.1%, 56.0%, 55.2%, and 55.2% (9, 10), respectively. On the other hand, the frequency of the *GSTT1* null genotype among Jordanian, Palestinian, Ashkenazi Jews and non-Ashkenazi Jews was 24.2%, 22.0%, 26.0%, 22.1% (9, 10), respectively. Comparisons between Iranian Mandaeans and above-mentioned populations demonstrating that Mandaeans showed higher and lower levels of the *GSTM1* and *GSTT1* null genotypes, respectively. There was remarkably difference between Mandaeans and other mentioned populations for the frequency of the *GSTM1* null genotype.

Mutation, gene flow and natural selection should be disregarded in interpretation the influence of evolutionary forces on Mandaeans and their surrounding gene pools. In Iran and Iraq Mandaeans lived as small and isolated ethno-religious communities. Therefore, genetic drift, at least in part might be account for differences between Mandaeans and other populations.

## References

[1] Hayes JD Strange RC (2000). Glutathione S-transferase polymorphisms and their biological consequences. Pharmacology, 61(3):154–66. 10971201 10.1159/000028396

[2] Khalighinasab MR Saify K Saadat M (2015). Association between null alleles of *GSTM1* and *GSTT1* and dependence to heroin and opium. Psychiatry Res, 228(3):977–8. 26003511 10.1016/j.psychres.2015.05.004

[3] Zendehboodi Z (2017). Association of glutathione S-transferase M1 and T1 polymorphisms and temperament. Mol Biol Res Commun, 6(3):95–100. 29071278 10.22099/mbrc.2017.4074 PMC5640891

[4] Rafiee L Saadat I Saadat M (2010). Glutathione S-transferase genetic polymorphisms (*GSTM1*, *GSTT1* and *GSTO2*) in three Iranian populations. Mol Biol Rep, 37(1):155–8. 19430957 10.1007/s11033-009-9565-8

[5] Nasseri G Zahedi T Mousavi-Kazerooni F Saadat M (2015). Prevalence of Null Genotypes of Glutathione S-Transferase T1 (*GSTT1*) and M1 (*GSTM1*) in Seven Iranian Populations. Iran J Public Health, 44(12):1655–61. 26811816 PMC4724738

[6] Amirshahi P Sunderland E Farhud DD (1992). Population genetics of the peoples of Iran I. Genetic polymorphisms of blood groups, serum proteins and red cell enzymes. Int J Anthropol, 7(3):1–10.

[7] Saadat M Zarghami M (2018). Consanguineous marriages among Iranian Mandaeans living in south-west Iran. J Biosoc Sci, 50(4):451–456. 28585514 10.1017/S0021932017000207

[8] Buckley JJ (2002). The Mandaeans: Ancient Texts and Modern People. First edition, Oxford University Press, New York.

[9] Karban A Krivoy N Elkin H (2011). Non-Jewish Israeli IBD patients have significantly higher glutathione S-transferase *GSTT1*-null frequency. Dig Dis Sci, 56(7):2081–7. 21243434 10.1007/s10620-010-1543-4

[10] Naffa RG Awidi AS Yousef AM Ismail SI (2012). CYP1AI, glutathione S transferase gene polymorphisms and risk of Polycythemia vera. Cancer Epidemiol, 36(1):68–72. 22018952 10.1016/j.canep.2011.05.001

